# Supplementary figures and images for: Localized semi-nonnegative matrix factorization (LocaNMF) of widefield calcium imaging data
Source: PLoS Comput Biol. 2020 Apr 13;16(4):e1007791. doi: 10.1371/journal.pcbi.1007791 (PMC7179949; doi:10.1371/journal.pcbi.1007791)

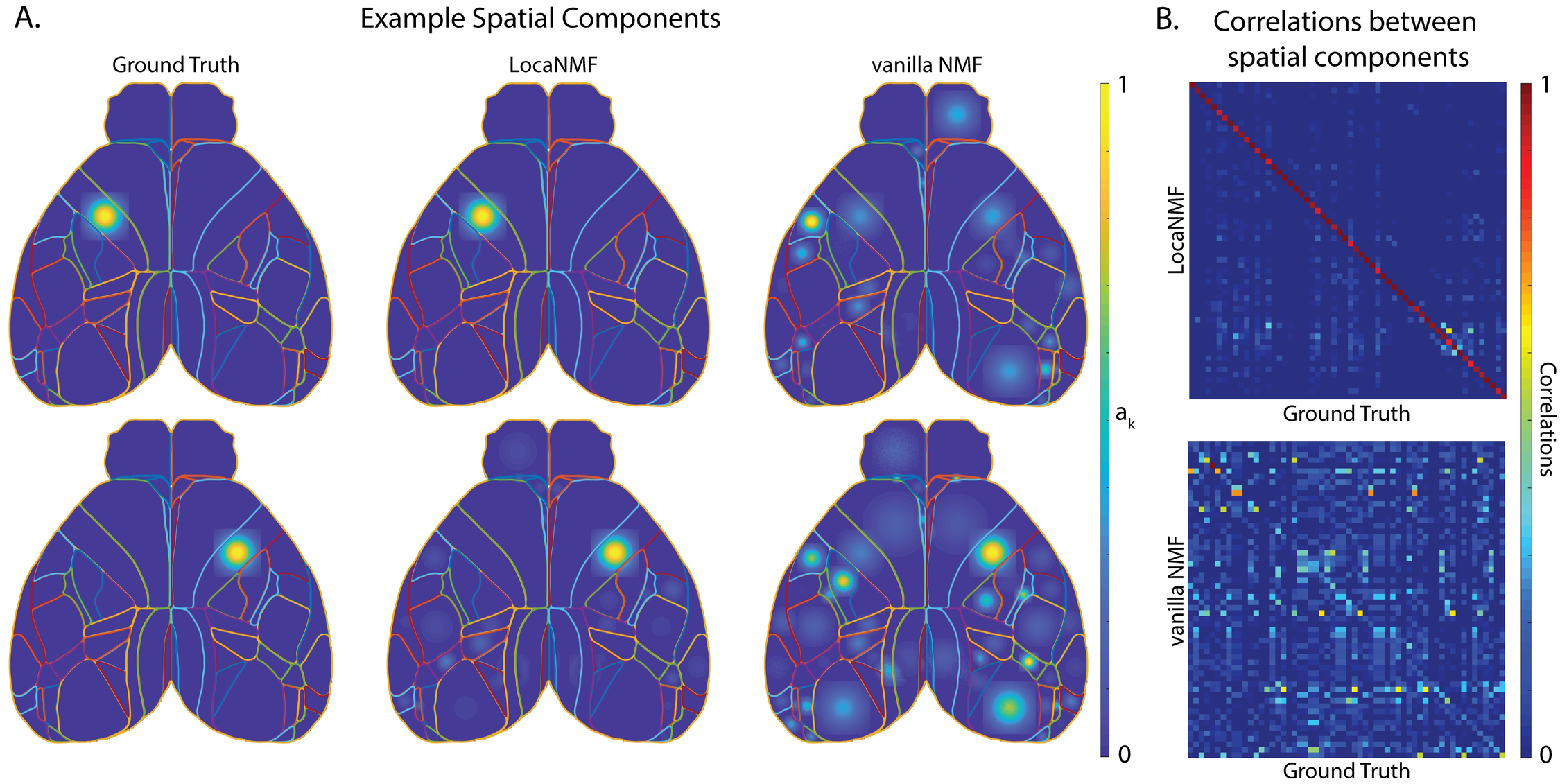

Supplement: S1 Fig — A-D. Legend and conclusions similar to Fig 2A–2D. (TIF) [file pcbi.1007791.s001.tif]

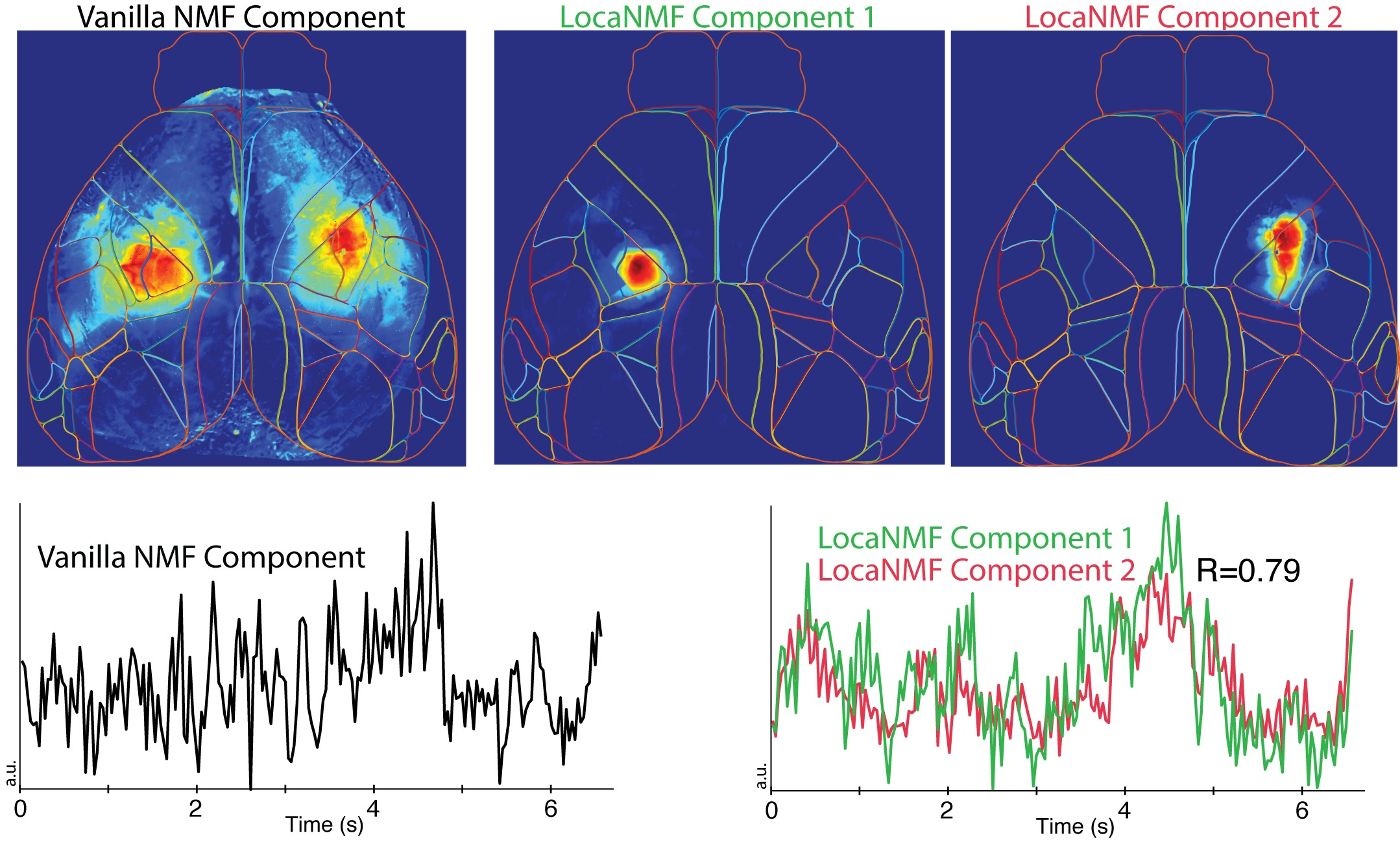

Supplement: S2 Fig — A-D. Legend and conclusions similar to Fig 5A–5D. (TIF) [file pcbi.1007791.s002.tif]

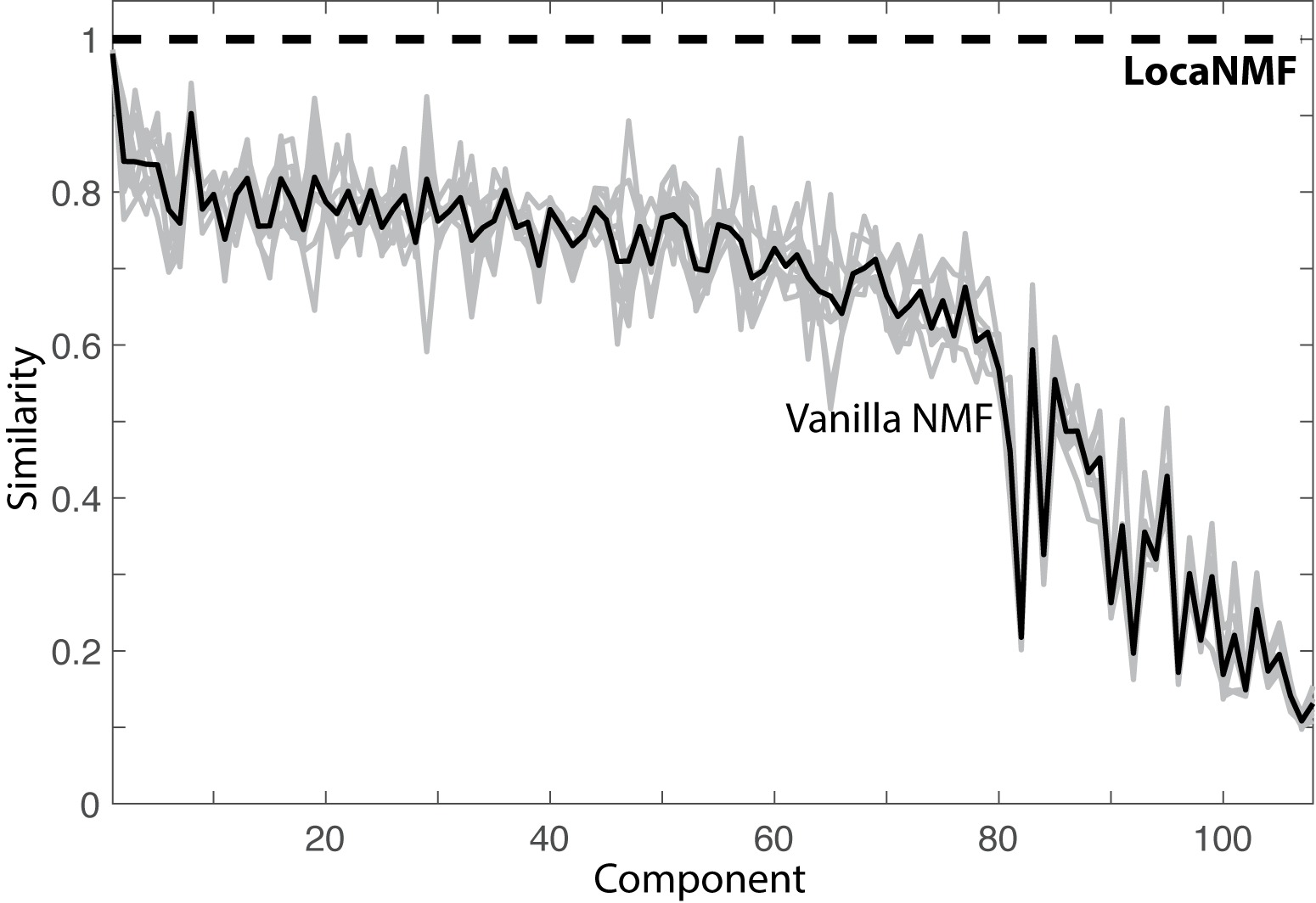

Supplement: S3 Fig — One of the randomly initialized vanilla NMF decomposition was chosen as the example decomposition, and each gray line shows the similarity between the components resulting from a different random initialization to this example decomposition, after component matching using a greedy search. The solid black line shows the mean similarity over initializations. The similarity across initializations is 1 for LocaNMF, shown here with a dashed black line. (TIF) [file pcbi.1007791.s003.tif]

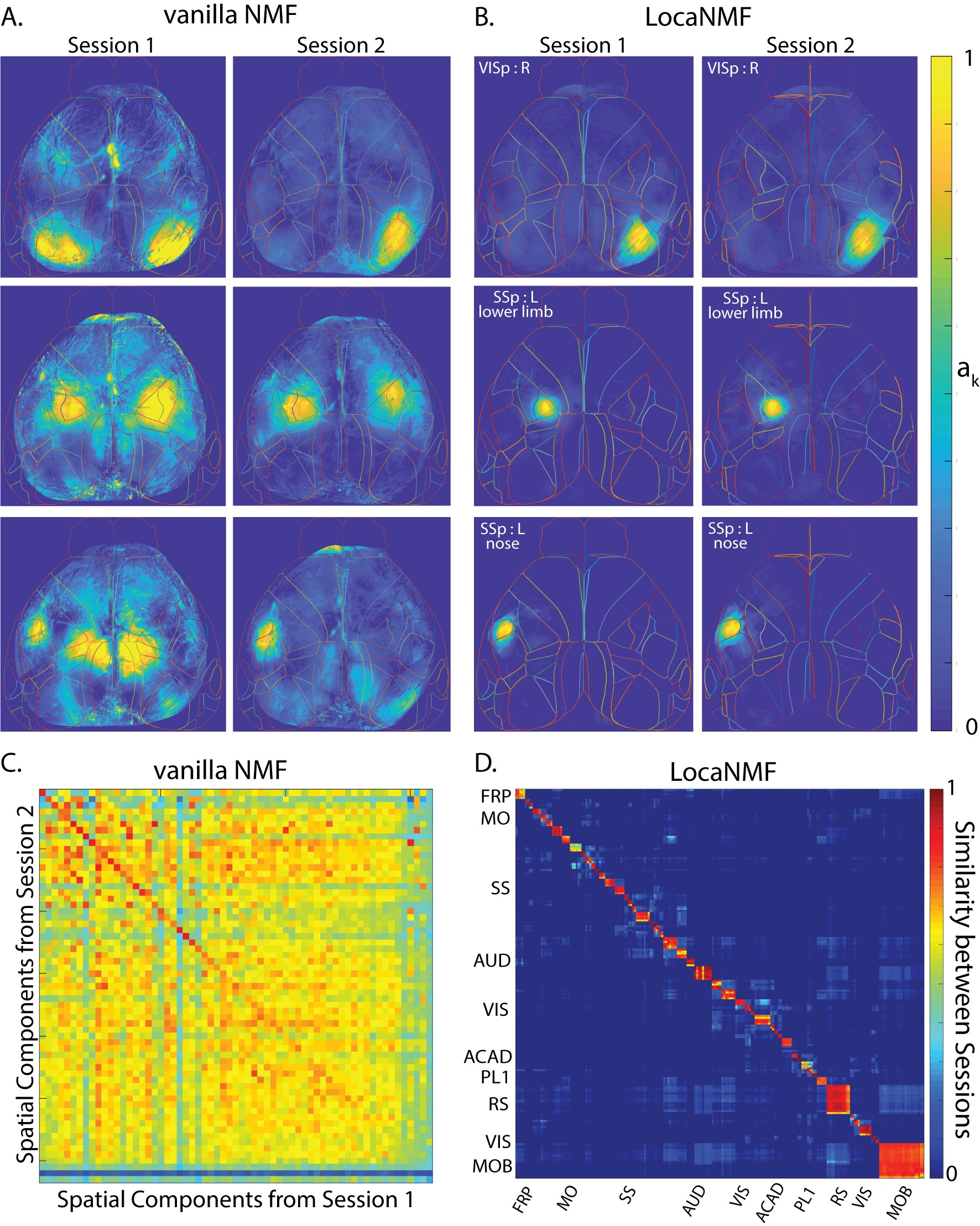

Supplement: S4 Fig — A-D. Legend and conclusions similar to Fig 6A–6D. (TIF) [file pcbi.1007791.s004.tif]

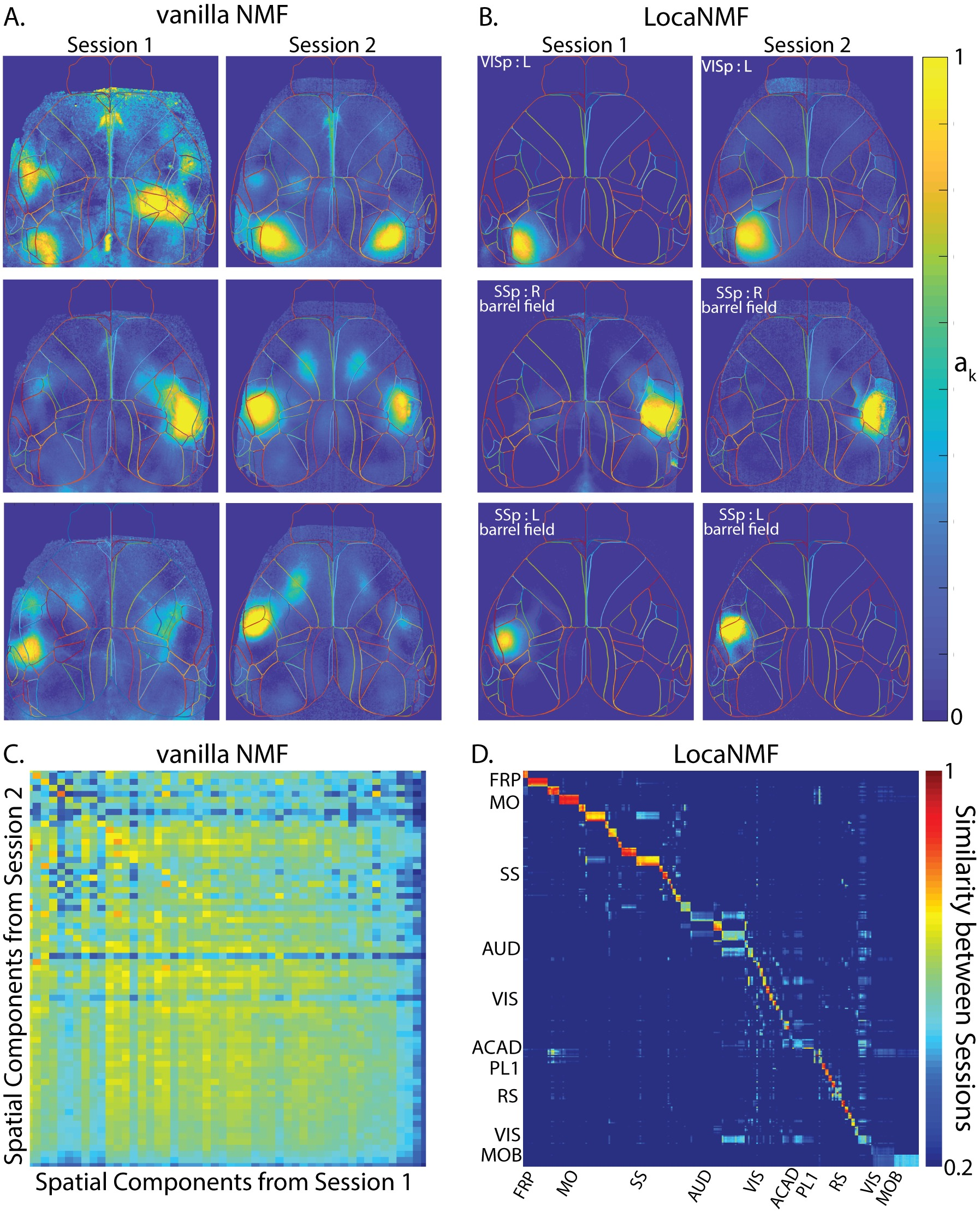

Supplement: S5 Fig — A-D. Legend and conclusions similar to Fig 7A–7D. (TIF) [file pcbi.1007791.s005.tif]

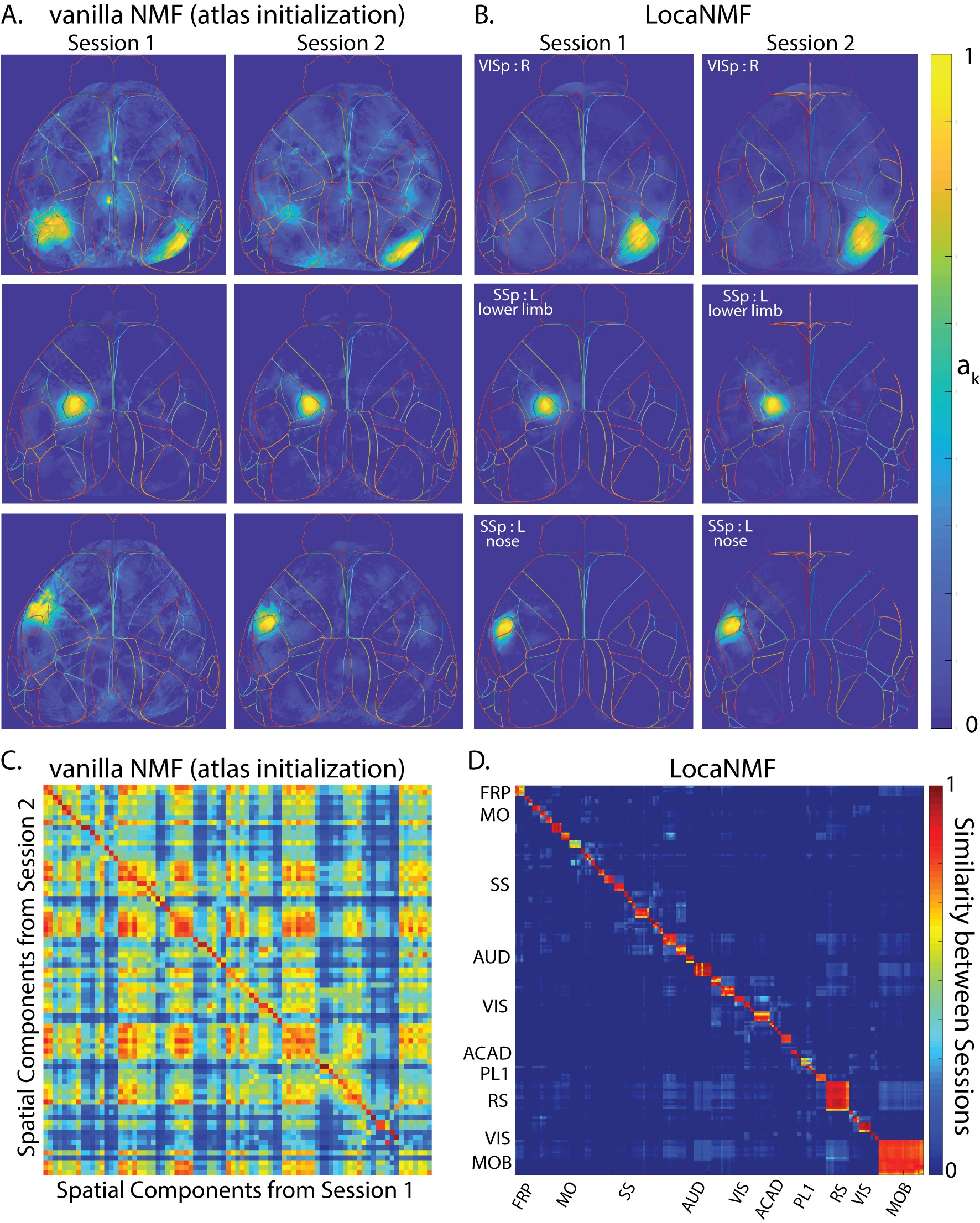

Supplement: S6 Fig — There is more stability across sessions as compared to S4A Fig, but LocaNMF provides more stability still due to the localization constraint. A-D. Legend and conclusions similar to Fig 6A–6D. (TIF) [file pcbi.1007791.s006.tif]

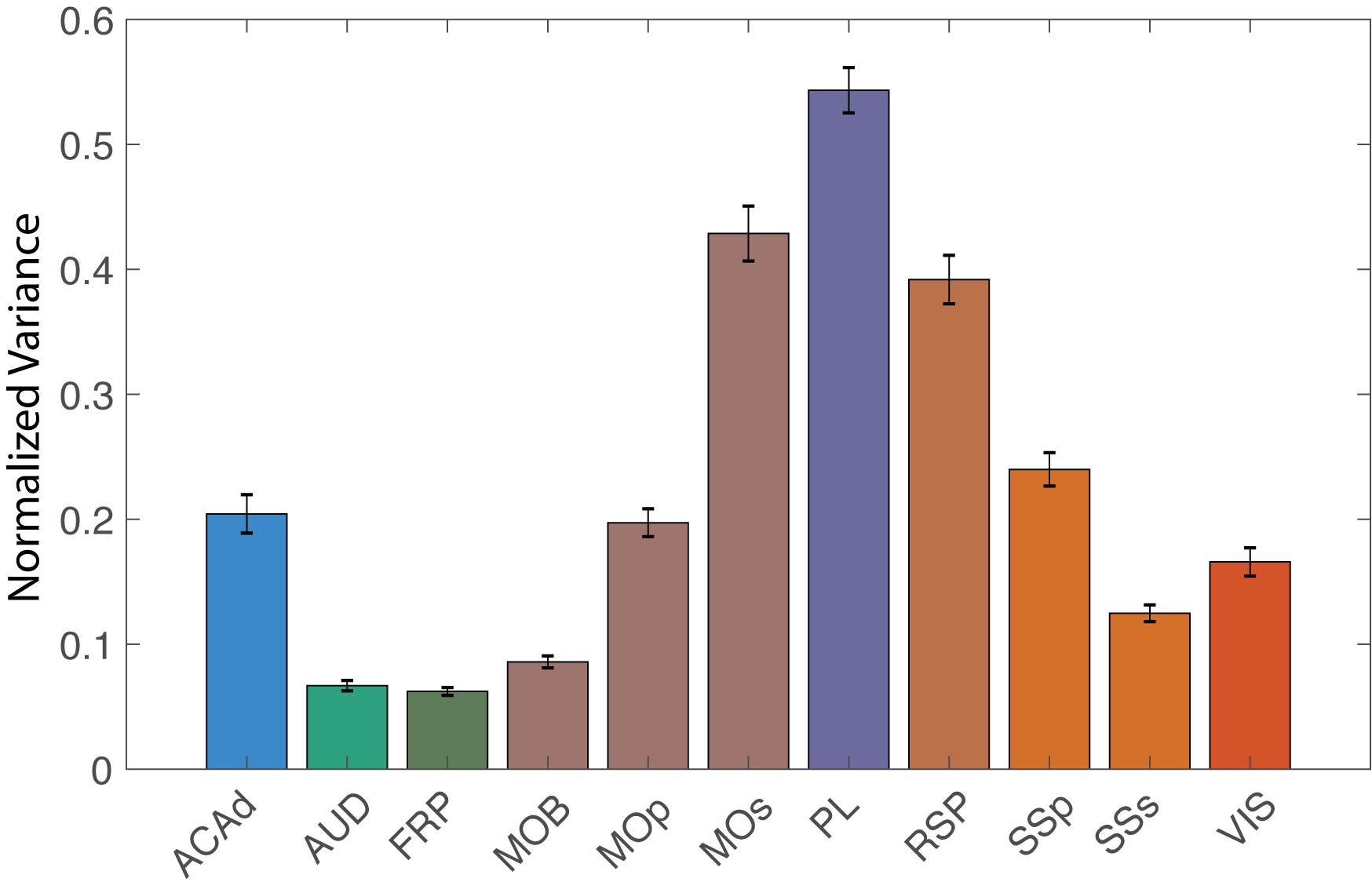

Supplement: S7 Fig — The values are normalized by the maximum variance in a particular session. The values shown here are means over sessions, for all 20 sessions in 10 mice in dataset 1, with the standard error of the mean depicted around the mean. (TIF) [file pcbi.1007791.s007.tif]
